# Wound-Healing Effects of Birch Bark and Propolis Extracts on Epidermolysis Bullosa Keratinocytes

**DOI:** 10.3390/ijms27135746

**Published:** 2026-06-25

**Authors:** Thomas Kissas, Dimitra Kiritsi, Ioannis Athanasiou, Alexander Nyström, Alexandros Onoufriadis, Ioannis Mourtzinos

**Affiliations:** 1 Department of Food Science and Technology, Faculty of Agriculture, Aristotle University of Thessaloniki, 54124 Thessaloniki, Greece; tkissas@agro.auth.gr; 2 Department of Dermatology, Faculty of Medicine, Medical Center—University of Freiburg, University of Freiburg, 79104 Freiburg, Germany or dimkyritsi@auth.gr (D.K.); ioannis.athanasiou@uniklinik-freiburg.de (I.A.); alexander.nystroem@uniklinik-freiburg.de (A.N.); 3 First Department of Dermatology, Faculty of Medicine, Aristotle University of Thessaloniki, 54124 Thessaloniki, Greece; 4 Laboratory of Medical Biology and Genetics, School of Medicine, Aristotle University of Thessaloniki, 54124 Thessaloniki, Greece; onoufriadis@auth.gr

**Keywords:** propolis, birch bark, delivery systems, wound-healing, scratch assay, epidermolysis bullosa

## Abstract

Epidermolysis bullosa (EB) is a group of genetic diseases characterized by skin fragility. Although therapeutic options aim to accelerate wound-healing, improvement is needed; therefore, birch bark and propolis were investigated due to their beneficial biological properties. A representative ethanolic extract was analyzed by reversed-phase high-performance liquid chromatography with diode array detection (RP-HPLC-DAD) for chemical profiling of the raw materials. A hydrophobic natural deep eutectic solvent (HNaDES) for birch bark extraction, as well as a hydrogel and a bigel enriched with propolis and birch bark extract, were prepared and characterized by Fourier transform infrared (FT-IR) spectroscopy. Cytotoxicity and wound-healing potential were evaluated using 3-(4,5-dimethylthiazol-2-yl)-2,5-diphenyltetrazolium bromide (MTT) and scratch assays in six human keratinocyte cell lines: two from healthy individuals, two from recessive dystrophic ΕΒ patients (RDEB), and two from laminin-332-deficient junctional EB patients (JEB). RP-HPLC-DAD revealed the presence of phenolic compounds (e.g., chrysin, pinocembrin, pinobanksin) and pentacyclic triterpenes (e.g., betulin and betulinic acid), characteristic of propolis and birch bark, respectively. FT-IR confirmed HNaDES formation and indicated physical interactions within the gels. All systems exhibited no cytotoxicity at 1 μg/mL and increased cell vitality. Moreover, in keratinocytes derived from JEB patients, hydrogel improved wound- healing significantly at 24 h, whereas bigel showed significant improvement at 8 h. The developed systems could be promising topical treatments.

## 1. Introduction

Epidermolysis bullosa (EB) is a group of genetic disorders characterized by the formation of blisters, erosions, and wounds of the skin and mucous membranes (eyes and esophagus) after minor mechanical injury [1]. EB is considered a rare disease; nevertheless, approximately 500,000 people are affected worldwide [2]. Based on the layer of skin in which the separation occurs and the subsequent formation of blisters, four types are distinguished, namely EB simplex (EBS), junctional EB (JEB), dystrophic EB (DEB), and Kindler EB (KEB). Until now, pathogenic variants in at least 20 genes encoding proteins essential for the integrity and adhesion of the skin layers have been associated with the different subtypes of the disease [3]. Patients with laminin-332-deficient JEB and DEB suffer from widespread, difficult-to-heal wounds; thus, improving wound-healing is a key therapeutic target [4]. Current therapeutic options aim to achieve accelerated wound-healing, but there is still scope for enhancement [5,6]. Furthermore, the recently approved re-dosable gene therapy is a rather costly procedure, which will not be available in large parts of the world.

Several in vivo studies, preclinical and clinical, evaluated birch bark extract’s or its active components’ ability to accelerate wound-healing [7,8,9]. Filsuvez^®^ is a topical gel containing birch bark extract, approved by both the European Medicines Agency (EMA) and Food and Drug Administration (FDA) for the treatment of partial-thickness wounds in patients aged ≥6 months old with DEB and JEB [10]. Another natural product with proven healing activity is propolis. Propolis is a complex mixture of substances released by bees and compounds derived from plants. The abundance of bioactive compounds in propolis enables it to exhibit antioxidant and antimicrobial activity, as well as the ability to accelerate wound-healing [11]. The latter is achieved by enhancing epithelial remodeling, regulating extracellular material deposition, and facilitating granular tissue formation [12].

To exploit the bioactive compounds of these materials, over the last few decades, researchers have mainly used organic solvents, such as hexane, ethanol, methanol, acetone, acetonitrile, and chloroform, associated with environmental pollution and toxicological concerns. Replacing conventional solvents with emerging, sustainable, non-toxic solvents, such as natural deep eutectic solvents (NaDESs), cyclodextrins, and edible oils, must be of primary importance. These solvents can effectively extract bioactive compounds and optimize drug delivery [13,14,15]. Moreover, the resulting extracts can be incorporated into biomaterials, like hydrogels, oleogels, and bigels, which not only exert properties as carriers but also contribute to healing [16].

The aim of the present study was to develop innovative, sustainable physicochemical delivery systems for the bioactive compounds found in birch bark and propolis. Furthermore, reversed-phase high-performance liquid chromatography with diode array detection (RP-HPLC-DAD) was employed for the characterization of raw materials, while Fourier transform infrared (FT-IR) spectroscopy was used to characterize both raw materials and formulations. Their cytotoxicity and wound-healing activity were subsequently assessed in vitro using keratinocyte cell lines derived from patients with EB.

## 2. Results and Discussion

### 2.1. RP-HPLC-DAD Analysis

The phenolic composition of the ethanolic extract obtained from birch bark and poplar-type propolis was analyzed by RP-HPLC–DAD. The identified compounds were quantified and expressed as mg/g dry weight of the plant materials. The results are shown in Table 1.

In the present study, flavonoids were found in higher concentrations than phenolic acids, with chrysin (1.895 mg/g), pinobanksin (1.784 mg/g), galangin (1.727 mg/g), and pinocembrin (1.512 mg/g) being the major constituents in the mixture of propolis and birch bark. The relatively low concentrations of phenolic acids observed are consistent with previously reported propolis profiles [17].

The flavonoid content is generally within the range reported in the literature for propolis samples from different geographical regions. In particular, the concentrations of chrysin, pinocembrin, caffeic acid phenethyl ester (CAPE), and quercetin were comparable to those reported for Polish and Uruguayan propolis samples [18]. However, phenolic acids were detected at lower levels compared to those reported in the same study. These differences may be attributed to variations in extraction conditions, including solvent composition, extraction time, and extraction procedure. Specifically, Kurek-Górecka et al. employed a two-step extraction of propolis using 70% ethanol (*w*/*v*), with each step lasting one week [18].

Moreover, the detected compounds have also been documented in Turkish propolis, including quercetin, galangin, pinocembrin, caffeic acid, p-coumaric acid, trans-ferulic acid, and CAPE, among others [19]. Nevertheless, quantitative differences were observed, further highlighting the chemical variability of propolis and its dependence on extraction conditions. In that study, a two-step extraction procedure using 70% ethanol was applied, with each step lasting 24 h [19]. Furthermore, propolis variability is influenced by botanical and geographical origin as well as by environmental conditions, such as seasonality and illumination [20].

Chrysin, galangin, pinocembrin, and CAPE are characteristic flavonoids of poplar-type propolis and contribute significantly to its antioxidant, anti-inflammatory, antimicrobial, and anticancer properties, while CAPE has also been associated with wound-healing effects [21,22].

Furthermore, HPLC analysis enabled the identification and quantification of the triterpenes betulin (1.625 mg/g) and betulinic acid (0.157 mg/g), which present a broad spectrum of biological properties, such as anti-inflammatory, antimicrobial, wound-healing, anticancer, and antioxidant effects [23,24]. The higher content of betulin compared to betulinic acid is consistent with previous studies [25,26]. These concentrations align with a study investigating birch bark extracts using a low transition temperature mixture (LTTM) as a solvent, where betulin and betulinic acid contents ranged from 0.491 to 1.788 mg/g and from 0.106 to 0.316 mg/g dry bark, respectively [26]. However, considerably higher contents of these compounds have also been reported in *Betula pendula* bark extracts depending on the botanical origin and extraction conditions employed [25,27].

### 2.2. Fourier Transform Infrared (FT-IR) Spectroscopy Characterization of the THY:LA (1:2)-ΒΒE Extract

**Figure 1:**
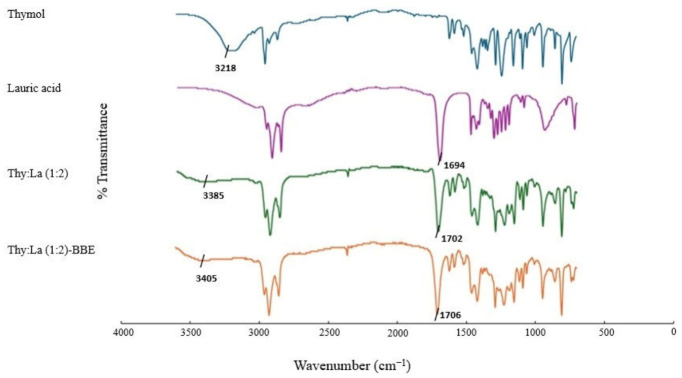
Fourier transform infrared (FT-IR) spectroscopy thymol, lauric acid, thymol:lauric acid at a 1:2 molar ratio (THY:LA (1:2)) as a hydrophobic natural deep eutectic solvent (HNaDES), and birch bark extract using thymol:lauric acid at a 1:2 molar ratio as a solvent (THY:LA (1:2)-ΒΒE extract). Key peaks are marked with black markers.

FT-IR analysis was employed to examine the molecular interactions between thymol and lauric acid in the THY:LA (1:2) HNaDES, as well as to characterize the resulting THY:LA (1:2)-ΒΒE extract (Figure 1).

In particular, the FT-IR spectrum of pure thymol showed a characteristic peak at 3218 cm^−1^ associated with the phenolic O-H bond. Thymol’s aromatic character is indicated by the C=C bond of the benzene ring at the two low-intensity peaks at 1620 cm^−1^ and 1585 cm^−1^ [28,29]. Lauric acid is another component of the prepared HNaDES. The compound exhibited peaks at 2912 cm^−1^ and 2847 cm^−1^, assigned to the stretching vibrations of the aliphatic CH_2_ (methylene) and CH_3_ (methyl) groups. Also, it showed a characteristic sharp peak at 1694 cm^−1^ associated with the carbonyl C=O bond stretching vibration [30].

Regarding the THY:LA (1:2) HNaDES, a shift in the O-H bond absorption of thymol from 3218 cm^−1^ to 3385 cm^−1^ and in the C=O bond of lauric acid from 1694 cm^−1^ to 1702 cm^−1^ indicated the formation of a hydrogen bonding network between the individual components, and thus the successful formation of the HNaDES [31].

As for the extract, it exhibited small shifts in higher wavenumbers compared to the solvent, indicating interaction of HNaDES with the extracted components. For example, a small shift from 1702 cm^−1^ to 1706 cm^−1^ was detected. Notably, a significant shift from 3385 cm^−1^ to 3405 cm^−1^ was also observed, which may be due to the interaction of thymol with the bioactive substances contained in birch bark. Similar FT-IR shifts have been reported for ginger extracts obtained using a NaDES comprising betaine/D,L-lactic acid/water (1:2:2.5) [32].

### 2.3. FT-IR Investigations of Oleogel, Hydrogel, and Bigel

**Figure 2:**
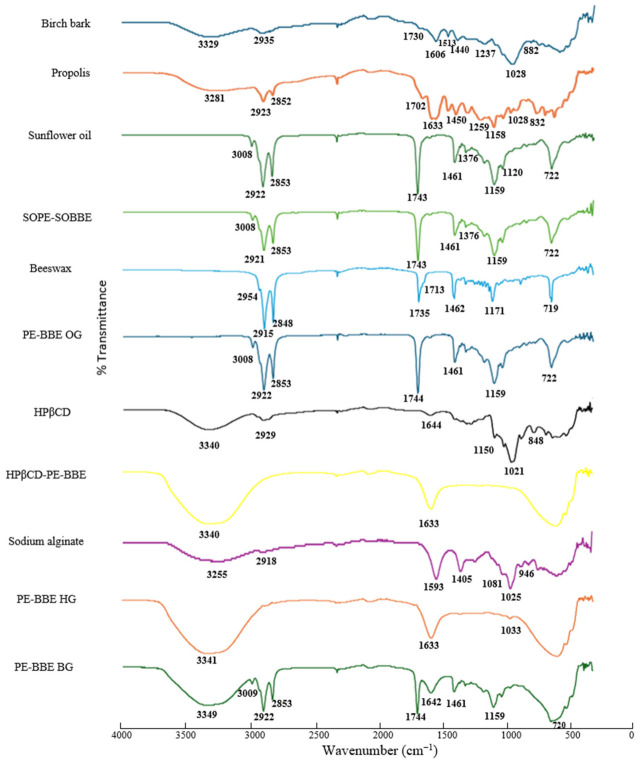
FT-IR spectra of birch bark, propolis, sunflower oil, propolis and birch bark sunflower oil extract (SOPE-SOBBE), beeswax, oleogel enriched with propolis and birch bark extract (PE-BBE OG), hydroxypropyl-β-cyclodextrin (HPβCD), propolis and birch bark aqueous solution of hydroxypropyl-β-cyclodextrin extract (HPβCD-PE-BBE), sodium alginate, hydrogel enriched with propolis and birch bark extract (PE-BBE HG), and bigel enriched with propolis and birch bark extract (PE-BBE BG).

To characterize the natural materials in the prepared formulations, spectra of both propolis and birch bark were included. Additionally, to study the formation mechanisms of the oleogel, hydrogel, and bigel, the FT-IR spectra of these and their constituents were also recorded (Figure 2).

FT-IR spectrum of birch bark revealed a broad absorption band at 3329 cm^−1^, corresponding to O-H stretching vibrations of alcohols and cellulose. Peaks at 2935 cm^−1^ and 1730 cm^−1^ were assigned to C-H asymmetric stretching and C=O ester bond stretching vibrations, respectively. Absorption bands in the 1500–1600 cm^−1^ were associated with aromatic ring vibrations of lignin, while the peak at 1448 cm^−1^ corresponded to aliphatic C-H bending vibrations. The peak at 1028 cm^−1^ was assigned to C-O stretching vibrations characteristic for cellulose, and it has been reported that lupeol and betulinic acid may contribute to this. The peak at 882 cm^−1^ is due to H-C-H wagging vibrations [33,34,35].

In the propolis extract, the peak at 3281 cm^−1^ was attributed to the hydroxyl groups of phenolic compounds. Peaks at 2923 cm^−1^ and 2852 cm^−1^ were attributed to asymmetric and symmetric stretching vibrations of aliphatic groups, respectively, while the peak at 1702 cm^−1^ is due to C=O ester bond vibrations. Peaks at 1633 cm^−1^ and 1603 cm^−1^ corresponded to C=C stretching vibrations of phenols. The C-H deformations and aromatic ring stretching vibrations at 1450 cm^−1^ were attributed to flavonoids. Additional bands at 1259 cm^−1^, 1158 cm^−1^, and 1029 cm^−1^ were associated with hydroxy flavonoids, esters associated with long-chain fatty acids, and phenolic -CH_2_OH groups, respectively. Finally, the peak at 832 cm^−1^ corresponded to the out-of-plane bending vibrations of the C-H bond, also found in phenols. These results align with previously published studies on propolis samples [36,37].

Sunflower oil showed a peak at 3008 cm^−1^ associated with cis=C-H stretching vibration. Strong peaks in the 2800–3000 cm^−1^ region corresponded to C-H stretching vibration, while the peaks at 1461 cm^−1^ and 1376 cm^−1^ are due to bending vibrations of CH_3_ and CH_2_ groups. In addition, a sharp peak is observed at 1743 cm^−1^, which is characteristic of oils with short hydrocarbon chains and saturated fatty acids, and is attributed to the C=O group stretching vibration. The peaks in the range of 1100–1200 cm^−1^ corresponded to the C-O ester bond stretching vibrations. Finally, the peak at 722 cm^−1^ may be due to the C-H bending vibrations of the CH_3_ group and alkanes [38,39]. Compared to the sunflower oil spectrum, the obtained SOPE-SOBBE extract showed no significant differences.

The beeswax spectrum showed peaks at 2954, 2915, and 2848 cm^−1^, attributed to CH_2_ and CH_3_ stretching vibrations. In addition, the scissoring vibrations and the rocking stretching vibrations of CH_2_ group corresponded to the peaks at 1462 cm^−1^ and 719 cm^−1^. Furthermore, the peak at 1735 cm^−1^ is attributed to the C=O stretching vibrations, and the peak at 1171 cm^−1^ corresponded to the C-H bending vibrations. The absorptions due to these bonds are characteristic of monoesters found in beeswax. Additionally, the absorption of fatty acids is recorded at 1713 cm^−1^. Similar beeswax spectra have been reported in the literature [40].

FT-IR spectrum of PE-BBE OG showed similar peaks to those observed in the sunflower oil spectrum, suggesting no chemical interactions between the plant materials and the oil. These results align with previous studies on oleogels enriched with different concentrations of birch bark extract particles [41].

HPβCD exhibited characteristic absorption bands due to saccharide at 3340 cm^−1^ (O-H stretching), 2929 cm^−1^ (C-H stretching), 1644 cm^−1^ (O-H bending), 1150 cm^−1^, and 1021 cm^−1^ (C-O stretching). In addition, a characteristic peak of the α-glycosidic bond was found at 848 cm^−1^ [42]. The aqueous extract of HPβCD exhibited peaks at 3340 cm^−1^ and 1633 cm^−1^, which were stronger than those shown in the HPβCD spectrum, and may be due to propolis and birch bark.

Sodium alginate exhibited a broad absorption band in the range of 3000–3600 cm^−1^, associated with O-H stretching vibrations. In addition, the absorption band between 2850 and 2920 cm^−1^ corresponded to the vibrations of the C-H aliphatic bond. The peaks at 1593 cm^−1^ and 1405 cm^−1^ were assigned to the symmetric and asymmetric stretching vibrations of carboxylic acids. The peak at 1083 cm^−1^ corresponded to the C-O-C stretching vibrations, and the peak at 1025 cm^−1^ was due to the C-O-H stretching vibrations. Both bonds occurred in the pyranozyl ring of sodium alginate. Finally, the peak at 946 cm^−1^ was due to the stretching vibration of the C-O bond, which is characteristic of uric acids [43].

PE-BBE HG showed distinctive peaks of HPβCD extract and sodium alginate, indicating that the hydrogel was developed without chemical interaction. Similar results have been reported in the literature [44].

Regarding the PE-BBE BG spectrum, the characteristic peaks of both oleogel and hydrogel were observed, without the appearance of new peaks or significant shifts, suggesting that the bigel formation is based on physical interactions rather than chemical bonding, which is in line with previous results [45].

### 2.4. Impact on Cell Vitality

**Figure 3:**
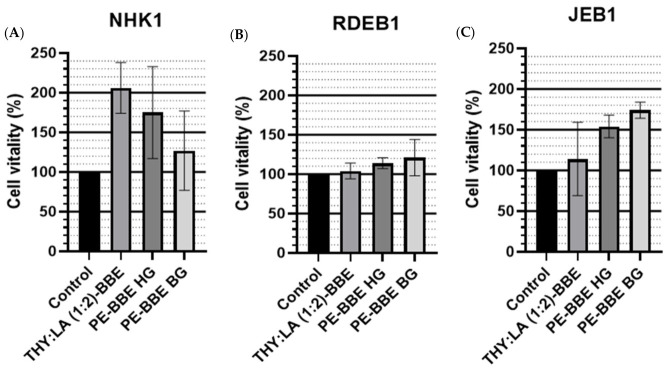

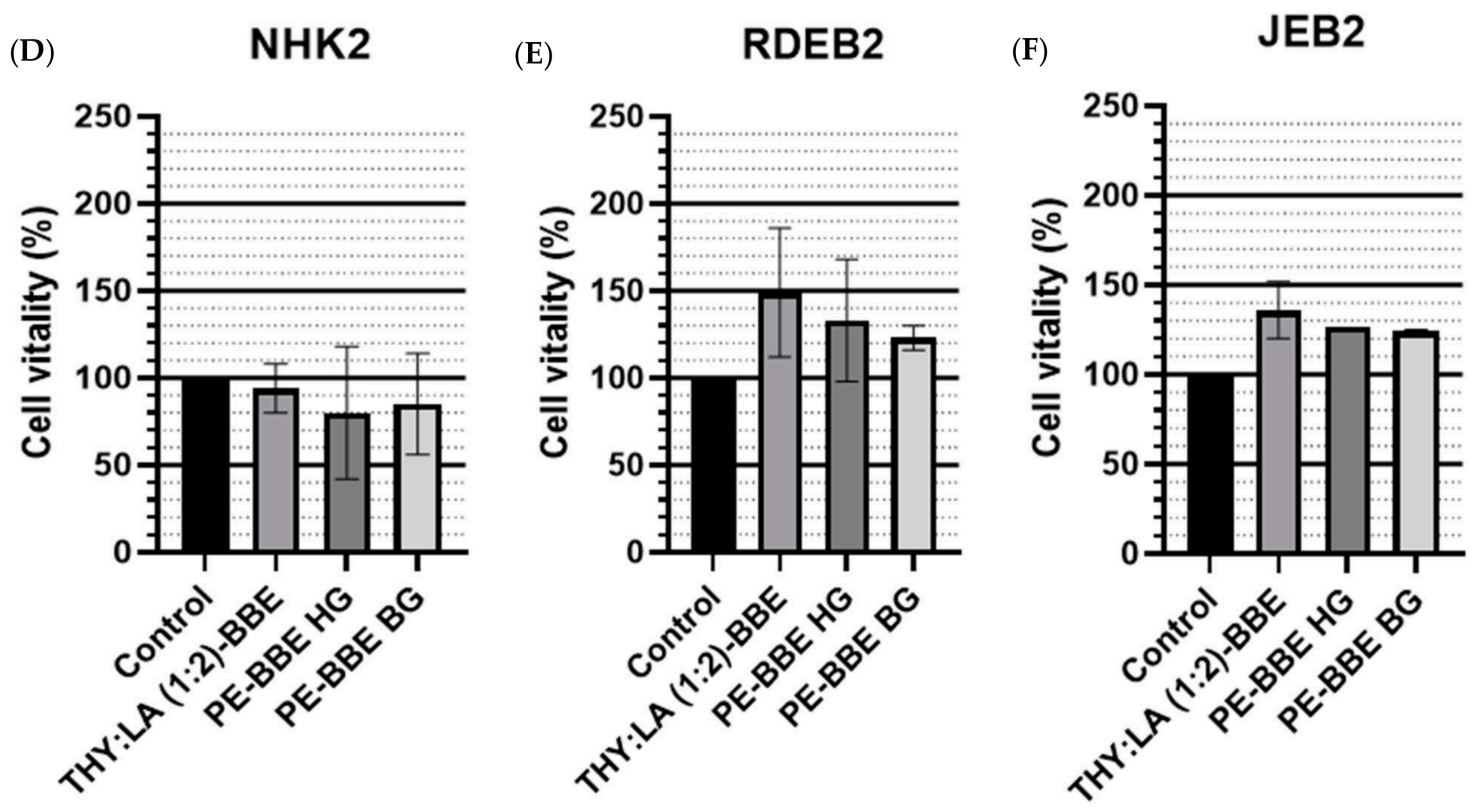
Cell vitality of normal human keratinocytes (NHK), NHK1 (**A**) and NHK2 (**D**), recessive dystrophic epidermolysis bullosa (RDEB) patient keratinocytes RDEB1 (**B**) and RDEB2 (**E**), and junctional epidermolysis bullosa (JEB) patient keratinocytes JEB1 (**C**) and JEB2 (**F**) following treatment with the physicochemical systems THY:LA (1:2)-BBE, PE-BBE HG, and PE-BBE BG. Cell vitality was determined by the 3-(4,5-dimethylthiazol-2-yl)-2,5-diphenyltetrazolium bromide (MTT) assay after 24 h of exposure to each system at a concentration of 1 μg/mL. Each bar graph represents % vitality. Control cells received only culture medium without treatment. Data were normalized to the untreated control, which is considered 100% viable. Experiments were performed in triplicate, and data are presented as mean ± SD. According to Tukey’s post hoc test, no statistically significant differences in cell vitality were observed among the treatment groups (*p*-value > 0.05).

The HNaDES birch bark extract and the hydrogel and bigel formulations containing birch bark and propolis extracts were initially tested for their effects on cell vitality. Testing cytotoxicity gives early insights regarding the toxicity of a biological material, which may help to evaluate its therapeutic relevance [46]. It has been reported that a compound or system is considered non-cytotoxic when cell vitality, assessed with an MTT assay, exceeds 80% [47]. Based on this assay, THY:LA (1:2)-BBE, PE-BBE HG, and PE-BBE BG demonstrated notable cell vitality, with levels consistently surpassing 80% when applied at a specific concentration of 1 µg/mL after 24 h of exposure on healthy human keratinocyte cell lines (NHK1 (A) and NHK2 (D)), as well as on EB patient cells; RDEB patient keratinocytes (RDEB1 (B) and RDEB2 (E)); and JEB patient keratinocytes (JEB1 (C) and JEB2 (F)) (Figure 3).

The cytotoxicity of birch bark has been investigated in normal human skin cells. Vater et al. reported that birch bark extract rich in betulin caused a mild increase in vitality in dermal fibroblasts, which could also be observed in epidermal keratinocytes [48]. A similar trend was recorded by other researchers, showing that betulin extract has no cytotoxicity in HaCaT cells [49]. Our findings are consistent with the aforementioned results.

The effect of propolis on cell vitality depends on factors such as its origin, concentration, and the type of cells to which it is applied [50]. In one study, the vitality of normal dermal fibroblasts improved with increasing concentration of ethanolic propolis extract [50]. Moreover, propolis extracts have demonstrated no cytotoxic effects in both macrophages (RAW 264.7 cells) and HaCaT keratinocytes, even after a long period of exposure [51]. In addition, other researchers found extremely low cytotoxicity for the tested propolis extracts on HaCaT keratinocytes [52,53]. These results suggest that propolis extract incorporation in the developed systems is not cytotoxic to keratinocytes, which was confirmed through the MTT assay.

Several studies have investigated oleogels or hydrogels enriched with birch bark or propolis regarding cell vitality. Oleogels with betulin and lupeol showed no toxicity to normal keratinocytes [54]. Similarly, incorporation of propolis into carbopol-based hydrogel had no adverse effect on NIH-3T3 fibroblast cells at a concentration up to 0.1 mg/mL [55]. Our findings suggest no cytotoxicity across all cell lines when applying hydrogels or bigels.

Although most of the components used in the preparation of NaDESs are naturally derived, the resulting solvent should not be regarded as non-toxic, and their toxicity must be evaluated [56]. In the present study, the HNaDES extract of birch bark did not affect cell vitality.

### 2.5. Effects on Scratch-Assay-Based Wound-Healing

A scratch assay was performed on keratinocyte monolayers to study the effects of THY:LA (1:2)-BBE, PE-BBE HG, and PE-BBE BG at a concentration of 1 μg/mL compared to the untreated control monolayer. This concentration was chosen as it was considered cytocompatible based on the previous MTT assay results.

In NHK1, complete healing was observed at 24 h with no statistical difference compared to the untreated cells, while in NHK2, all systems significantly enhanced healing at 8 h. Accelerating the wound-healing process helps prevent complications, such as wound infections, and contributes to a better long-term outcome [7]. Regarding the RDEB1 and RDEB2 lines, it is noteworthy that complete wound-healing (97–100%) was observed as early as 8 h, though differences were not statistically significant.

Laminin-332-deficient JEB keratinocytes, when compared to RDEB keratinocytes, displayed inherently impaired wound-healing. Consequently, the clearest effects on wound-healing were observed in JEB keratinocytes. In the JEB1 line, PE-BBE HG improved healing significantly at 24 h (100%) while, in JEB2 cells, PE-BBE BG yielded 34% wound closure at 8 h, which was significantly higher than the untreated control cells. Both JEB lines had markedly lower wound closure percentages at 8 h compared to the other lines. These findings align with clinical data evaluating the efficacy of Filsuvez^®^, showing a lower rate of complete healing on day 45 in JEB patients (18.6%) compared to those with RDEB (44.4%) [57]. Nevertheless, the fact that treatment with bigel and hydrogel significantly surpassed the wound-healing percentage of untreated cells at 8 h and 24 h, respectively, may suggest that these systems contribute better to this type of EB.

**Figure 4:**
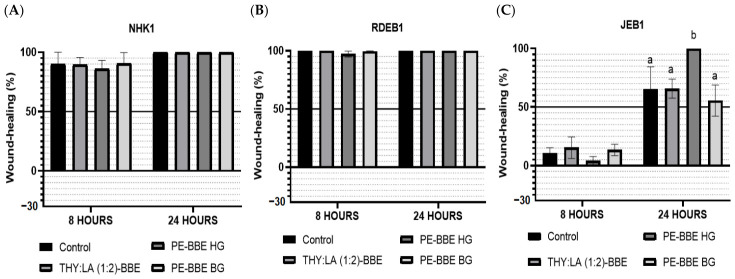

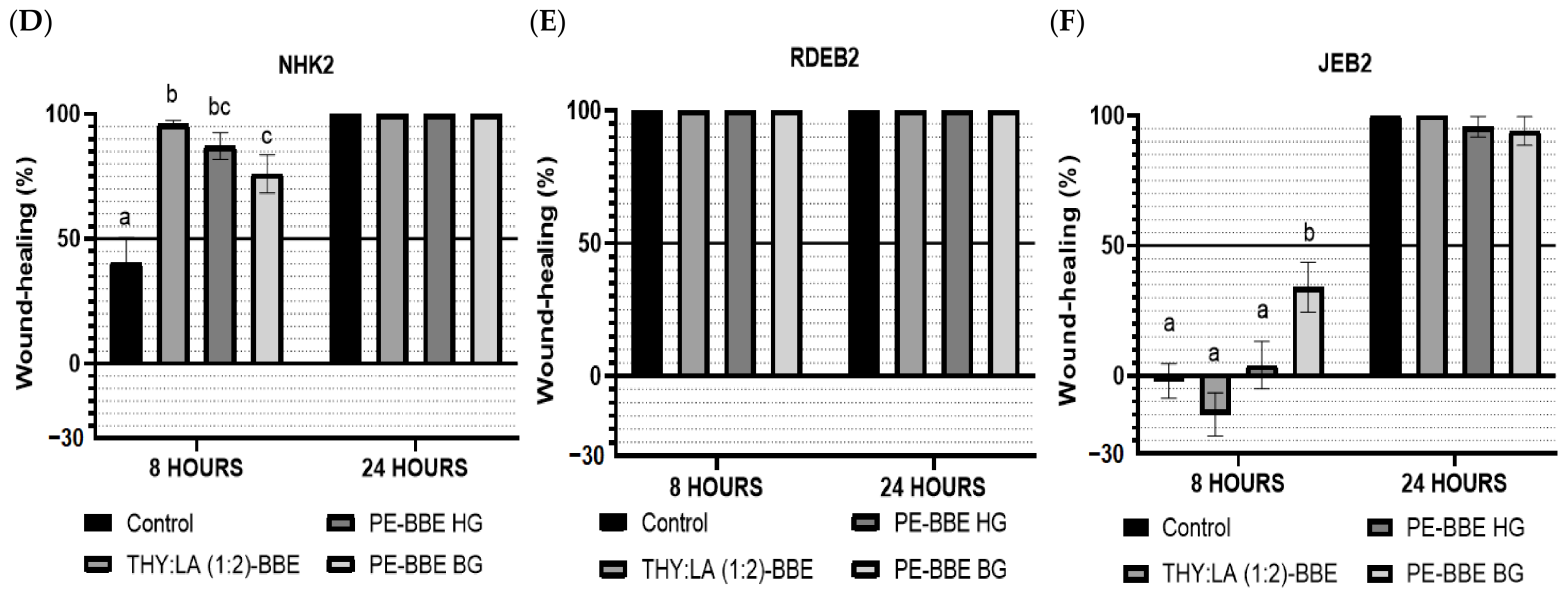
Percentage of wound closure in scratch assays after exposure to THY:LA (1:2)-BBE, PE-BBE HG, and PE-BBE BG at 8 h and 24 h. The three physicochemical systems were applied on normal human keratinocytes NHK1 (**A**) and NHK2 (**D**), on RDEB patient keratinocytes RDEB1 (**B**) and RDEB2 (**E**), and on JEB patient keratinocytes JEB1 (**C**) and JEB2 (**F**) at a concentration of 1 μg/mL. Experiments were performed in triplicate, and data are presented as mean ± SD. Different letters indicate statistically significant differences among groups, according to Tukey’s post hoc test (*p* < 0.05).

Overall, the physicochemical systems showed similar effects within the different cell lines, and no significant differences were observed among them, with two exceptions. In the NHK2 cell line at 8 h, the THY:LA (1:2)-ΒΒE extract significantly enhanced wound-healing compared to PE-BBE BG, whereas in the JEB1 cell line at 8 h, PE-BBE HG demonstrated significantly higher wound healing (%) compared to the other two systems (Figure 4).

**Figure 5:**
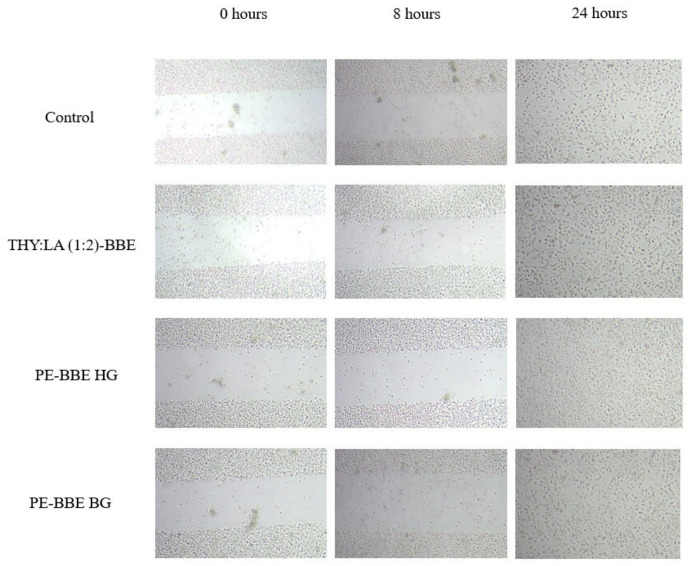
Representative microscope images of wound areas in JEB1 cells at 0, 8, and 24 h after application of THY:LA (1:2)-BBE, PE-BBE HG, and PE-BBE BG. Images were taken at 4× magnification of the objective lens. Scale bar = 100 μm. Control cells received only culture medium without treatment.

The complete wound healing (%) dataset for all cell lines and physicochemical systems at 8 h and 24 h is provided in the Supplementary Materials (Table S1). An example of an in vitro scratch assay after treatment of JEB1 with all physicochemical systems can be seen in Figure 5.

Until now, the combination of propolis and birch bark extracts has not been investigated. However, the observed positive effect of the developed hydrogel and bigel formulations could be evidence of a potential additive or synergistic interaction between the bioactive compounds present in both materials.

Birch bark exerts its wound-healing properties by exhibiting antioxidant activity, enhanced keratinocyte migration (possibly through stimulation of cytoskeletal reorganization, including actin filaments and stress fibers) and proliferation, collagen deposition, and regulation of inflammatory and growth factors. These mechanisms collectively accelerate wound contraction and re-epithelialization. This beneficial activity is primarily attributed to its pentacyclic triterpenes, especially betulin, along with betulinic acid, oleanolic acid, erythrodiol, and lupeol [58,59]. Betulin has been shown to interact with mitochondrial membranes, altering their surface properties and modulating mitochondrial function [60]. Given the emerging role of mitochondrial function and mitophagy in regulating keratinocyte migration and proliferation during wound repair [61], these membranotropic effects may contribute, at least in part, to the pleiotropic wound-healing activity of birch bark triterpenes.

The wound-healing effects of propolis are attributed to its antimicrobial, anti-inflammatory, and antioxidant effects. In addition, it has been shown to support keratinocyte proliferation, migration, and differentiation and to enhance angiogenesis via upregulation of VEGF. Propolis also modulates inflammatory responses by regulating cytokines, such as IL-1, IL-6, and TNF, contributing to improved tissue regeneration. Through these activities, propolis appears to shorten healing time, reduce scar formation, aid in wound shrinkage, and, ultimately, improve the quality of life for patients. These properties are primarily due to its polyphenolic flavonoids [11,62,63].

The biological activities reported for birch bark and propolis provide a mechanistic rationale for wound-healing; however, the underlying molecular mechanisms were not investigated in the present study and are discussed based on the existing literature.

Several studies have investigated the wound-healing potential of birch bark and propolis extracts. Ebeling et al. found that triterpene extract and betulin increased keratinocyte migration [59]. In contrast, propolis exhibits variable effects on cell migration depending on its botanical origin, composition, and concentration, ranging from stimulatory [64] to no significant effect [53,65].

Bigel enriched with birch bark and propolis extracts was investigated for the first time. As pharmaceutical structures, bigels exhibit great interest since they combine the advantages of hydrogels and oleogels. In the present study, the oleogel was based on propolis and birch bark extracts, with sunflower oil as the solvent and beeswax serving as the oleogelator. In a previous study, sunflower oil, in combination with birch bark extract, significantly improved healing. It was concluded that the oil used as a carrier affects wound-healing not only by regulating the release of the extract but also through its biological action [13]. Furthermore, sunflower oil can serve as a barrier against oxygen, delaying the oxidation of bioactive compounds [66].

On the other hand, the hydrogel consisted of aqueous HPβCD extract of propolis and birch bark, with sodium alginate serving as the hydrogelator. While hydrogels are widely used for drug delivery, they often show poor entrapment and a rapid, non-linear release of hydrophobic compounds. Cyclodextrins can mitigate these issues by enhancing swelling capacity, drug retention, and release time of the drug from the hydrogels [67]. Although simultaneous incorporation of propolis and birch bark extract into hydrogels has not been reported, propolis-based hydrogels have shown promising healing activity [68,69]. Both the hydrogel and bigel systems in the present study exhibited positive wound-healing effects.

Lastly, the positive effect shown by the HNaDES systems aligns with findings from previous studies. Specifically, NaDESs, with their increased solubilization capacity and adjustable selectivity, have been considered advantageous in extraction processes, especially for the stabilization of labile metabolites [70]. Moreover, extracts obtained using NaDESs have been reported to preserve or even enhance the biological activity of the bioactive compounds that are dissolved, although further studies are needed [71]. To the best of our knowledge, DES systems containing birch bark and/or propolis have not previously been evaluated in the literature for their effects in scratch assay tests.

## 3. Materials and Methods

### 3.1. Reagents

Thymol (>99%) was purchased from TCI (Tokyo, Japan), whereas lauric acid (≥98%) was from Sigma-Aldrich (Saint Louis, MO, USA). HPβCD was from Gangwal Chemicals Pvt. Ltd. (Maharashtra, Mumbai, India), white beeswax was obtained from CHEMCO (Athens, Greece), and sodium alginate from ThermoFisher GmbH (Kandel, Germany), while sunflower oil (Vita D’or) was purchased from a local market (Thessaloniki, Greece). Keratinocyte Serum-Free Media (K-SFM) and DPBS were from Gibco (Paisley, UK), while trypsin/EDTA was purchased from PAN Biotech GmbH (Aidenbach, Germany). FBS, DMSO, and Mitomycin-C were from Sigma-Aldrich (Saint Louis, MO, USA). MTT reagent was from ATCC bioproducts (Manassas, VA, USA).

### 3.2. Standards and Solvents

Betulin (≥98%), chrysin (≥99%), ferulic acid (≥98%), pinobanksin (≥95%), pinocembrin (≥98%), and galangin (≥99%) were all purchased from Extrasynthese (Genay, France). Betulinic acid (≥98%), caffeic acid (≥98%), quercetin (≥95%), and rutin (≥94%) were from Sigma-Aldrich (St. Louis, MO, USA), while caffeic acid phenethyl ester (>98%) was from TCI (Tokyo, Japan). p-Coumaric acid (≥98%) was obtained from Biosynth Carbosynth (Bratislava, Slovakia). HPLC-grade methanol (MeOH) and water (H_2_O) were acquired from Chem-Lab (Zedelgem, Belgium). Acetonitrile was purchased from VWR Chemicals (Rosny-sous-Bois, France) and 85% phosphoric acid was obtained from Sordalab (Étampes, France).

### 3.3. Plant Materials

Commercial dried and shredded birch bark product (*Betula pendula*, Betulaceae) (AgoraMarket, Patras, Greece; origin: Poland) was ground and stored at room temperature. Raw propolis (Lustrel Laboratoires S.A., Saint Jean De Vedas, Siret, France) was also ground and stored at −20 °C. According to the product’s specifications, the purified propolis originated from China from the resin of poplar buds (*Populus* spp.) harvested by bees. It had a total flavonoid content *>* 7.0%, and its color ranged from brown to black.

### 3.4. Preparation of Physicochemical Delivery Systems

#### 3.4.1. HNaDES Preparation and Extraction Procedure

The HNaDES solvent was prepared by mixing appropriate amounts of terpene thymol as the hydrogen bond acceptor (HBA) and a fatty acid namely lauric acid as the hydrogen bond donor (HBD) at a 1:2 molar ratio (THY:LA (1:2)). The mixture was heated at 50 °C, and mixing occurred at 750 rpm until a transparent, homogeneous liquid formed, as reported by Kyriakoudi et al. [31]. After cooling, the HNaDES was used the same day or stored at room temperature for further use. For extraction, birch bark (5 g) was mixed with 50 mL of THY:LA (1:2) to obtain a solid/liquid ratio of 1:10 (*w*/*v*) using a magnetic stirrer (600 rpm) for 1 h at 60 °C (THY:LA (1:2)-BBE).

#### 3.4.2. Sunflower Oil Extraction

Sunflower oil, as a vegetable oil, was used as a sustainable solvent for the recovery of hydrophobic compounds. Birch bark (2.5 g) and propolis (2.5 g) were mixed with 50 mL of sunflower oil to obtain a solid-to-liquid ratio of 1:10 (*w*/*v*) using a magnetic stirrer (600 rpm) for 1 h at 60 °C. The obtained mixture was centrifuged at 5000× *g* for 6 min. The supernatant was filtered and stored at −20 °C for further study (SOPE-SOBBE).

#### 3.4.3. Oleogel Preparation

An oleogel (PE-BBE OG) was developed as a part of the subsequently prepared bigel. The extract of propolis and birch bark, using sunflower oil as the solvent and white beeswax (3% *w*/*w*) as the oleogelator, were dissolved under continuous mechanical stirring (430 rpm) and heated at 70 °C for 15 min [72], then cooled at room temperature for 40 min [73] and stored at 4 °C for at least 24 h before being used for the bigel preparation [74].

#### 3.4.4. Extraction with Cyclodextrin Aqueous Solution

Aqueous solution of HPβCD was used as an alternative hydrophilic carrier. Birch bark (2.5 g) and propolis (2.5 g) were mixed with HPβCD aqueous solution (C_HPβCD_ = 10 mg/mL), at a solid-to-liquid ratio of 1:10 (*w*/*v*). The mixture was subjected to extraction under stirring at 460 rpm for 1 h in a water bath at 60 °C, then centrifuged at 5000× *g* for 6 min. The supernatant was filtered and stored at −20 °C for further study (HPβCD-PE-BBE).

#### 3.4.5. Hydrogel Preparation

For hydrogel preparation (PE-BBE HG), the extract of propolis and birch bark, using HPβCD aqueous solution as the solvent and sodium alginate (3% *w*/*w*) as the hydrogelator, were weighed and solubilized under continuous mechanical stirring (890 rpm) for 15 min at room temperature. The hydrogels were stored at 4 °C for at least 24 h before being used for the bigel preparation [74].

#### 3.4.6. Bigel Preparation

Bigel formulation (PE-BBE BG) was prepared by adding the hydrogel to the oleogel at a molar ratio of 20:80 using mechanical stirring (800 rpm) for 10 min at room temperature. The bigel was then stored at 4 °C [74].

### 3.5. RP-HPLC-DAD Analysis of Birch Bark and Propolis Ethanolic Extract

Before HPLC analysis, a representative ethanolic extract was prepared. Birch bark (2.5 g) and propolis (2.5 g) were mixed with 50 mL of 80% (*v*/*v*) aqueous ethanol to obtain a solid-to-liquid ratio of 1:10 (*w*/*v*) using a magnetic stirrer (600 rpm) for 1 h at 60 °C. The obtained mixture was centrifuged at 5000× *g* for 6 min. The supernatant was filtered and stored at −20 °C for further analysis.

Phenolic compounds in the propolis and birch bark ethanolic extract were determined using HPLC, as described by Wu et al. [75], with some modifications. The analysis was carried out using an HPLC system consisting of an Agilent 1260 Infinity II Quaternary Pump VL, an Agilent 1260 Infinity II Autosampler, and an Agilent 1260 Infinity II Diode Array Detector High Sensitivity. Separation was carried out on a FORTIS C18 (250 mm × 4.6 mm i.d., 5 µm) reversed-phase column (Fortis Technologies Ltd., Neston, UK) at ambient temperature. The mobile phase consisted of an aqueous–phosphoric acid solution (0.1%, *v*/*v*) (A) and acetonitrile (B). The elution protocol was as follows: 0 min, 5% B; 0–10 min, 10% B; 10–30 min, 20% B; 30–50 min, 45% B; 50–70 min, 60% B; and 70–80 min, 5% B. The total run time was 80 min with a flow rate of 1 mL/min. The injection volume was 10 μL. Monitoring was in the range of 190–400 nm.

Pentacyclic triterpenes (betulin and betulinic acid) were analyzed under isocratic conditions according to Zhao et al. [76], with slight modifications. The mobile phase consisted of acetonitrile–water (86:14, *v*/*v*) with a flow rate of 1.0 mL/min and injection volume was 10 μL. Detection was performed at *λ* = 210 nm. The total run time was 25 min.

Compounds were identified by comparing retention times and UV spectra with those of reference standards. The extract was analyzed after proper dilution with methanol and filtration through 0.45 μm hydrophilic PTFE filters (Frisenette, Knebel, Denmark). Chromatographic data were processed using the OpenLab CDS version 3.5 software (2021, Agilent Technologies, Santa Clara, CA, USA).

Stock standard solutions (1000 μg/mL) were prepared using HPLC-grade methanol and stored at −20 °C till further use. Calibration curves for quantitative analysis were constructed using a minimum of five concentration levels (*n* = 5). All calibration curves showed good linearity (R^2^ > 0.994) for the analyzed compounds, including phenolic compounds (e.g., caffeic acid, caffeic acid phenethyl ester, chrysin, galangin, ferulic acid, p-coumaric acid, pinobanksin, pinocembrin, quercetin, rutin) and pentacyclic triterpenes (betulin and betulinic acid). The linearity, correlation coefficient (R^2^), retention time, and detection wavelength of the analyzed compounds are presented in Table 2.

### 3.6. FT-IR Spectroscopy

FT-IR spectroscopy was employed to study the molecular interactions within HNaDES components and their extract, as well as to investigate the formation mechanism of the enriched gels. FT-IR spectra were recorded using an FT-IR 6700 spectrometer (JASCO, Great Dunmow, UK) equipped with a MIRacle ™-Universal ATR sampling accessory (Pike Technologies, Madison, WI, USA) with a 3-Reflection Diamond/ZnSe Performance Crystal Plate. The samples were applied to cover the entire surface of the diamond crystal prism. The spectral data were the results of 64 scans, with 4 cm^−1^ resolution, covering a range of 400–4000 cm^−1^ in transmittance mode. Three spectra were recorded for each sample, and their average was used for further processing. The background signal (air) was recorded before each measurement. The spectra were processed using Spectra Manager software (V.2.15.01, JASCO, Great Dunmow, UK). Peaks between 700 and 3600 cm^−1^ were analyzed to investigate the prepared HNaDES.

### 3.7. Cell Culture

Studies were performed on two keratinocyte lines from healthy donors (NHK1 & NHK2); two from RDEB patients (RDEB1 & RDEB2; for both, diagnosis was confirmed by immunofluorescence mapping and genetic analyses, both lines were complete collagen VII negative by Western blotting); and two from patients suffering from laminin-332-deficient JEB (JEB1 & JEB2; for both, diagnosis was confirmed by immunofluorescence mapping and genetic analyses, both donors had pathogenic variants of laminin beta 3 and laminin-332 was strongly reduced by Western blotting). All cell lines were provided from the laboratory of the Department of Dermatology, Medical Center—University of Freiburg, Freiburg. Keratinocytes were immortalized with E6/E7, as previously described [77]. The study was approved by the Ethics Committee at Freiburg University (# 521/13). Cells were cultured in K-SFM at 37 °C with 5% CO_2_.

### 3.8. Cell Vitality and Proliferation Assay

A cell vitality and proliferation assay was undertaken to evaluate the cytocompatibility of the physicochemical systems following the ATCC protocol [78]. Initially, the systems were diluted in DPBS or DMSO, sterilized using 0.22 μm filter (Merck, Cork, Ireland), and stock solutions were prepared at a concentration of 10 mg/mL. Keratinocytes were seeded in 96-well plates (10^3^ cells/well) and treated with 1 µg/mL of each system for 24 h. This concentration was selected based on previously reported cytocompatible ranges for birch bark triterpene formulations [79]. After incubation, 10 μL of MTT reagent was added, and the cells were incubated for 4 h until a visible purple formazan precipitate formed. Subsequently, 100 μL of detergent was added, and the plates were kept in the dark for 2 h. Absorbance was measured at 570 nm on a microplate reader. Results were compared to control cells treated with culture medium alone, which was defined as 100% vitality. The percentage of cell vitality was calculated using the following Equation:

Cell vitality (%) = (mean absorbance of sample/mean absorbance of control) × 100(1)

### 3.9. Scratch Assay

Scratch assays were implemented to investigate the wound-healing effects of the systems and followed the Martinotti and Ranzato protocol [80]. Each system was diluted in culture medium and applied at 1 µg/mL, with untreated wells serving as controls. Each cell line was cultured at 37 °C with 5% CO_2_ until the confluent monolayer reached approximately 90%, then scratched with a 200 µL pipette tip and rinsed with PBS to remove detached cells and cell waste. Images were captured at 0, 8, and 24 h using a ZEISS Axiocam 208 color camera (ZEISS, Jena, Germany) at 4× magnification. Image analysis was performed using ImageJ software (version 1.54, National Institutes of Health, Bethesda, MD, USA). The following Equation was used to calculate the wound-healing rate [81]:

Wound-healing % = (A_t=0h_ − A_t=Δh_/A_t=0h_) × 100 (2)

A_t=0h_ is the wound area measured immediately after wound infliction.

A_t=Δh_ is the wound area measured hours, h, after wound infliction.

### 3.10. Statistical Analysis

The results were performed in triplicate, and the data were statistically analyzed using IBM SPSS Statistics 29 (IBM, Armonk, NY, USA). A one-way analysis of variance (one-way ANOVA) followed by Tukey’s post hoc test were performed to compare the controls with the cells to which the physicochemical systems were applied, as well as to evaluate differences among the physicochemical systems. The statistical significance (*p*) level was set at 5% (0.05).

## 4. Conclusions

Our findings indicate that keratinocytes treated with all the developed formulations exhibited enhanced cell vitality. Moreover, PE-BBE HG and PE-BBE BG displayed higher percentage wound closures in scratch assays than the control, especially in the cell lines derived from JEB patients. The use of such easily accessible and generally well-tolerated plant-based compounds could be beneficial not just for individuals with EB worldwide but also potentially for patients suffering from poor wound-healing in the general population. Further in-depth studies on their underlying molecular mechanisms and clinical trials are necessary to validate these findings.

## Figures and Tables

**Table 1:** Reversed phase high-performance liquid chromatography with diode array detection (RP-HPLC-DAD) quantification in ethanolic extract of propolis and birch bark.

Compounds	Ethanolic Extract of Propolis and Birch Bark
	mg/g of Plant Materials
Betulin	1.695 ± 0.115
Betulinic acid	0.157 ± 0.023
Caffeic acid	0.015 ± 0.001
Caffeic acid phenethyl ester	0.390 ± 0.001
Chrysin	1.895 ± 0.002
Galangin	1.727 ± 0.032
Ferulic acid	0.029 ± 0.001
p-Coumaric acid	0.073 ± 0.001
Pinobanksin	1.784 ± 0.002
Pinocembrin	1.512 ± 0.003
Quercetin	0.097 ± 0.001
Rutin	0.110 ± 0.001

Results are expressed as mean value ± SD of triplicate experiments (*n* = 3).

**Table 2:** Linearity, correlation coefficient (R^2^), retention time, and detection wavelength of 10 phenolic compounds and two pentacyclic triterpenes.

Compound	Linearity Range (μg/mL)	Linearity Equation	R^2^	Retention Time/min	Detection Wavelength/nm
Betulin	700–50	y ^a^ = 3.5482x ^b^ + 25.369	0.9969	12.41	210
Betulinic acid	250–25	y = 32,224x + 81,768	0.9996	9.19	210
Caffeic acid	250–1	y = 37.19x − 28.091	0.9981	21.14	323
Caffeic acid phenethyl ester	500–5	y = 24.101x − 75.021	0.9998	57.67	323
Chrysin	500–5	y = 49.609x + 319.23	0.9994	56.94	268
Galangin	500–5	y = 22.5x − 22.795	0.9998	58.62	266
Ferulic acid	500–5	y = 40.353x − 34.063	0.9998	31.50	323
p-Coumaric acid	250–10	y = 57.701x − 157.51	0.9982	28.43	310
Pinobanksin	500–5	y = 26.523x − 41.698	0.9997	48.15	292
Pinocembrin	500–5	y = 38.553x + 10.37	1.0000	57.19	290
Quercetin	250–10	y = 13.439x − 3.9925	0.9987	44.38	372
Rutin	250–10	y = 6.5186x − 38.072	0.9971	32.88	360

^a^ peak area; ^b^ concentration of standard (μg/mL).

## Data Availability

The original contributions presented in this study are included in the article/Supplementary Materials. Further inquiries can by directed to the corresponding author.

## Supplementary Materials

The following supporting information can be downloaded at: https://www.mdpi.com/article/10.3390/ijms27135746/s1.

## Abbreviations

The following abbreviations are used in this manuscript:

BBE	Birch bark extract
CAPE	Caffeic acid phenethyl ester
DDEB	Dominant dystrophic epidermolysis bullosa
FTIR	Fourier transform infrared spectroscopy
HNaDES	Hydrophobic natural deep eutectic solvent
HPβCD	Hydroxypropyl-β-cyclodextrin
IL	Interleukin
JEB	Junctional epidermolysis bullosa
MTT	3-(4,5-dimethylthiazol-2-yl)-2,5-diphenyltetrazolium bromide
NHK	Normal human keratinocytes
PE	Propolis extract
RDEB	Recessive dystrophic epidermolysis bullosa
RP-HPLC-DAD	Reversed-phase high-performance liquid chromatography–diode array detector
TNF	Tumor necrosis factor
VEGF	Vascular endothelial growth factor
