# Supplementary material for: Wound-Healing Effects of Birch Bark and Propolis Extracts on Epidermolysis Bullosa Keratinocytes
Source: Int J Mol Sci. 2026 Jun 25;27(13):5746. doi: 10.3390/ijms27135746 (PMC13361687; doi:10.3390/ijms27135746)
Supplement: Supplementary file 1 [file ijms-27-05746-s001.zip › ijms-4376721-supplementary.pdf]

Table S1. Quantitative wound-healing (%) data for all cell lines and physicochemical systems applied at 8 h and 24 h.

Table S1 Mean wound healing (%) values for all experimental conditions across the six keratinocyte cell lines (NHK1, NHK2, RDEB1, RDEB2, JEB1, and JEB2) at 8 h and 24 h. Values were calculated relative to the initial wound area (0 h). Data are presented for each physicochemical system (THY:LA (1:2)-BBE, PE-BBE HG, and PE-BBE BG) and the untreated control group. Data are expressed as mean ± SD. Different letters indicate statistically significant differences among treatments within each cell line according to Tukey's post hoc test ($p < 0.05$).

Wound-healing (%) NHK1		
	8 HOURS	24 HOURS
Control	90,0 ± 10	100,0 ± 0,0
THY:LA (1:2)-BBE	89,6 ± 6,0	100,0 ± 0,0
PE-BBE HG	86,1 ± 7,1	100,0 ± 0,0
PE-BBE BG	90,7 ± 9,0	100,0 ± 0,0
Wound-healing (%) NHK2		
	8 HOURS	24 HOURS
Control	$40,4^{a}$ ± 10,0	100,0 ± 0,0
THY:LA (1:2)-BBE	$96,1^{b}$ ± 1,4	100,0 ± 0,0
PE-BBE HG	$87,2^{bc}$ ± 5,4	100,0 ± 0,0
PE-BBE BG	$76,0^{c}$ ± 7,7	100,0 ± 0,0
Wound-healing (%) RDEB1		
	8 HOURS	24 HOURS
Control	100,0 ± 0,0	100,0 ± 0,0
THY:LA (1:2)-BBE	100,0 ± 0,0	100,0 ± 0,0
PE-BBE HG	97,1 ± 2,6	100,0 ± 0,0
PE-BBE BG	99,4 ± 0,5	100,0 ± 0,0
Wound-healing (%) RDEB2		
	8 HOURS	24 HOURS
Control	100,0 ± 0,0	100,0 ± 0,0
THY:LA (1:2)-BBE	100,0 ± 0,0	100,0 ± 0,0
PE-BBE HG	100,0 ± 0,0	100,0 ± 0,0
PE-BBE BG	100,0 ± 0,0	100,0 ± 0,0
Wound-healing (%) JEB1		
	8 HOURS	24 HOURS
Control	10,2 ± 5,0	$65,3^{a}$ ± 19,0
THY:LA (1:2)-BBE	15,2 ± 9,2	$65,7^{a}$ ± 8,1
PE-BBE HG	4,1 ± 3,6	$100,0^{b}$ ± 0,0
PE-BBE BG	13,3 ± 4,8	$55,4^{a}$ ± 13,3
Wound-healing (%) JEB2		
	8 HOURS	24 HOURS
Control	$-2,1^{a}$ ± 6,6	100,0 ± 0,0
THY:LA (1:2)-BBE	$-14,9^{a}$ ± 8,3	100,0 ± 0,0
PE-BBE HG	$4,0^{a}$ ± 9,1	95,7 ± 4,0
PE-BBE BG	$34,0^{b}$ ± 9,6	94,1 ± 5,5
